# 

**Published:** 1892-07-09

**Authors:** 


No. 302. Vol. XII.] SATURDAY, JULY 9th, 1892. [TWOPENCE.

